# Chitosan Woven Meshes: Influence of Threads Configuration on Mechanical, Morphological, and Physiological Properties

**DOI:** 10.3390/polym13010047

**Published:** 2020-12-25

**Authors:** Henrique Nunes da Silva, Milena Costa da Silva, Flavia Suzany Ferreira dos Santos, José Alberto Campos da Silva Júnior, Rossemberg Cardoso Barbosa, Marcus Vinícius Lia Fook

**Affiliations:** 1 Postgraduate Program in Materials Science and Engineering, Department of Materials Engineering, Federal University of Campina Grande, Campina Grande, PB 58429-900, Brazil; henrique.nunes.silva.eng@gmail.com (H.N.d.S.); milecost@hotmail.com (M.C.d.S.); flaviasuzanyfs@gmail.com (F.S.F.d.S.); albertocampos007@gmail.com (J.A.C.d.S.J.); 2 Department of Materials Engineering, Federal University of Campina Grande, Campina Grande, PB 58429-900, Brazil; rcbvet@gmail.com

**Keywords:** chitosan, wet-spinning, threads, weaving, woven mesh, biotextiles

## Abstract

This study aimed to develop meshes from the weaving of mono- and multifilament wet-spun chitosan (CS), for possible biomedical applications. In the wet-spinning process, CS solution (4% *w*/*v*) was extruded in a coagulation bath containing 70% sodium hydroxide solution (0.5 M), and 30% methanol was used. The multifilament thread was prepared by twisted of two and three monofilaments. CS threads obtained were characterized by tensile tests and scanning electron microscopy (SEM). Moreover, it was verified from the morphological tests that threads preserve the characteristics of the individual filaments and present typical “skin-core” microstructure obtained by wet spinning. CS woven meshes obtained were evaluated by optical microscopy (OM), tensile test, swelling degree, and in vitro enzymatic biodegradation. Mechanical properties, biodegradation rate, and amount of fluid absorbed of CS woven meshes were influenced by thread configuration. Hydrated CS meshes showed a larger elastic zone than the dry state. Therefore, CS woven meshes were obtained with modular properties from thread configuration used in weaving, suggesting potential applications in the biomedical field, like dressings, controlled drug delivery systems, or mechanical support.

## 1. Introduction

Chitosan (CS) is a natural, semi-crystalline linear polysaccharide, derived from chitin. Its chemical structure is composed of units of glucosamine and *N*-acetyl-glucosamine linked by glycosidic bonds β-(1 → 4). This polymer is insoluble in aqueous solutions with a pH > 7; however, in diluted acids (pH < 6), the free amine groups protonated in glucosamine facilitate the solubility of the polymer molecule. CS is a non-toxic, biodegradable, bioabsorbable polymer, and its degradation products are non-carcinogenic, non-immunogenic and its inflammatory reactions are minimal; properties that justify the variety of studies for biomedical applications [1,2,3,4]. Besides, CS can form films and filaments after the solubilization [5,6,7].

CS filaments can be obtained by the wet spinning technique, through the extrusion of polymeric solutions in a non-solvent, involving an acid–base neutralization reaction [8,9]. These filaments have the potential to be processed to obtain fibrous textile structures, such as fabrics (or woven meshes), knits (or meshes), braids, and nonwovens, which may have architecture designed to meet the specific requirements for applications in the biomedical field, such as dressings, reinforcement in the treatment of hernias, supports for cell growth in tissue engineering or even controlled drug delivery systems [10,11,12,13]. In this sense, among the techniques for obtaining textile structures, weaving stands out for making it possible to obtain woven meshes with various possible constructions, from specific textiles projects [10,14].

Textile biomaterials find applications in tissue engineering [15,16], wound healing [17], and mechanical support for hernia repair [11]. In this sense, research was carried out on the use of biodegradable fibrous structures such as scaffolds, dressings, dermal grafts, and controlled drug delivery systems. Almeida et al. [18] developed mesh structures based on polybutylene and silk fibroin for tissue engineering applications. Wang et al. [17] evaluated the application of a poly(lactic acid-co-glycolic acid) coated with a CS-collagen blend to correct skin defects in rats. You et al. [15] studied the application of collagen-coated poly(L-lactic acid-co-glycolic acid) (PLGA) mesh as dermal grafts in rats. Wang et al. [19] developed a CS-collagen mesh reinforced with a polyurethane mesh potential for skin tissue engineering. Wawro et al. [20] developed knitted fabrics from multifilament CS threads containing nanoparticles of silver, platinum, copper, and gold, the product obtained presented itself as a promising material for medical use, such as dressings, scaffolds, and surgical meshes. Aghaei-Ghareh-Bolagh et al. [14] developed woven meshes using manual weaving of electro-spun multifilament threads of tropoelastin and silk fibroin.

To the best of our knowledge, studies about CS woven meshes have not been reported. Therefore, the present work aimed to develop and characterize CS woven meshes from the weaving of mono- and multifilament threads, wet-spun and twisted, for possible biomedical applications. The properties of CS filaments and woven meshes are studied.

## 2. Materials and Methods

### 2.1. Materials

Chitosan from the exoskeleton of *Lipopenaeus vannamei* shrimp with Mv = 310 kDa, determined by viscometry (PSL Rheotek, São Paulo, Brazil), and degree of deacetylation of 86%, determined by the infrared spectroscopy (Perkin Elmer, Beaconsfield, UK) by the method by Brugnerotto et al. [21] were produced in the Northeastern Biomaterials Evaluation and Development Laboratory—CERTBIO (Campina Grande, PB, Brazil). Lactic acid 85% and methanol were obtained from Anidrol^®^ (Diadema, SP, Brazil). Sodium hydroxide (NaOH) was purchased from Neon^®^ (São Paulo, SP, Brazil). Phosphate buffer saline (PBS, pH = 7.4) and lysozyme (from chicken egg white, specific activity, Shugar units > 23000 U/mg) were acquired from Sigma Aldrich^®^, Merck Group (Darmstadt, Germany).

### 2.2. Chitosan Threads Preparation

**Figure 1:**
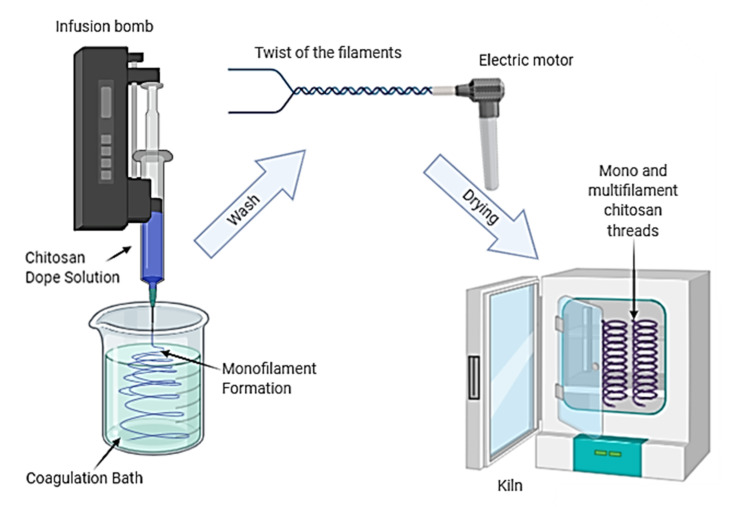
Schematic representing the preparation of the chitosan mono- and multifilament threads by wet-spinning followed by twisting.

CS solution (4% *w*/*v*) was prepared as previously reported [22,23,24]. In short, CS was dissolved in a lactic acid aqueous solution (0.206 mol/L), in stoichiometric relationship with CS amine groups, at constant stirring (200 rpm) at 25 ± 1 °C for 2 h. The obtained CS solution was transferred to a syringe (20 mL capacity and 1 mm diameter outlet tip) for subsequent wet spinning. CS solution was extruded in the coagulation bath (70% of 0.5 M sodium hydroxide (NaOH) and 30% methanol) at a flow rate of 45 mL/h using an infusion pump (Pump 11 Pico Plus Elite, Harvard Apparatus, Holliston, MA, USA) at 25 ± 1 °C. Finally, the filaments were taken out, washed with distilled water until wash water reaches a pH close to 7. Multifilament CS threads were prepared by the twist of two or three wets spun monofilaments using an electric motor (RW 20 D, IKA, Campinas, SP, Brazil) (at 80 rpm), as Figure 1 shows. The length of monofilaments was fixed at 2 m and the torsion time was 90 s. Mono- and multifilament CS threads were subsequently kiln-dried at 60 ± 2 °C for 1.5 h.

### 2.3. Chitosan Woven Meshes Preparation

**Figure 2:**
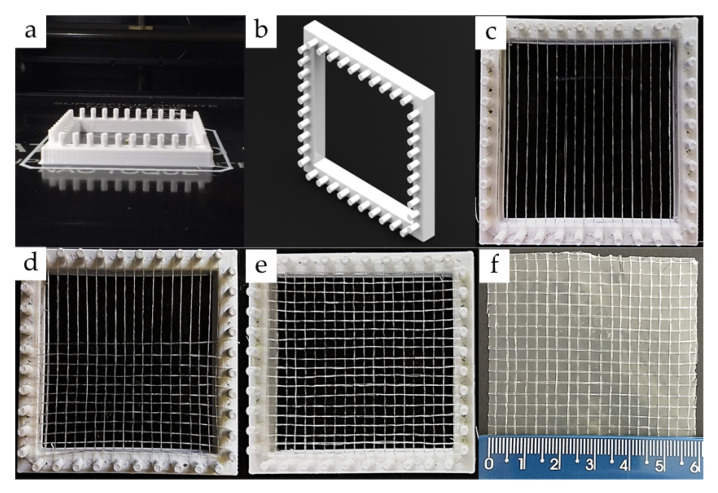
Preparation of the chitosan woven meshes from the weaving of chitosan threads: (**a**,**b**) views of the mold made by 3D printing; (**c**) the first plane of threads forming the textile warp; (**d**) secund plane of threads orthogonally inserted into the warp forming a plain weft (1/1); (**e**) chitosan woven meshes obtained by weaving, and (**f**) final chitosan woven mesh coated with a 1.5% chitosan solution.

A mold made by 3D printing (glycol-modified poly(ethylene terephthalate)—PETG), (3D Cloner, model DH PLUS, Marechal Cândido Rondon, PR, Brazil) was used to obtain the CS woven meshes (Figure 2). Diameter and spacing between mold pins were 3 mm and the edge was 6 mm (Figure 2a,b).

At beginning of weaving the CS threads (composed of one, two, and three filaments) were arranged in parallel to form a textile warp (Figure 2c). Next, a second CS threads plane was orthogonally inserted into warp forming a plain weft (1/1), as noted in Figure 2d,e. The opening of CS woven mesh (#) was defined by the spacing and diameter of mold pins, as well as CS threads diameter (Figure 2e). After weaving, CS woven meshes were coated with a 1.5% CS solution (dissolved in lactic acid solution 0.077 M), neutralized in 0.5 M NaOH solution (for 30 min), and dried at 50 ± 2 °C for 6 h. Woven mesh coating had the function to maintain the orthogonal relationship and weave between CS threads (Figure 2f).

### 2.4. Optical Microscopy (OM)

CS woven meshes were evaluated at a Hirox Digital Optical Microscope (Kh 1300 M, Tokyo, Japan). The measurements were performed using ImageJ (Java 1.8.0.112 Version, National Institutes of Health and Laboratory for Optical and Computational Instrumentation, Wisconsin, WI, USA) software directly on images obtained. ImageJ software to estimated CS woven meshes parameters.

### 2.5. Scanning Electron Microscopy (SEM)

Surface and cross-section morphology of mono- and multifilament CS threads was evaluated using a Scanning Electron Microscope Phenom World, model Pro-X800-07334 (Eindhoven, The Netherlands). The samples suffered cryogenic fracture followed by coating with a thin layer of gold. Images were taken by applying an electron beam accelerating voltage of 15 kV, with a depth of focus of 1 mm and a resolution of 30 nm. Measurements (*n* = 10) of CS threads diameter, pore size, and twist angle were made using ImageJ software.

### 2.6. Mechanical Properties

Uniaxial tensile testing was used to characterize the mechanical properties of CS treads (*n* = 10), dry and hydrated CS woven meshes (*n* = 5). Samples with 100 mm long to threads and 10 × 60 mm^2^ to woven meshes were analyzed. The CS threads tests were performed using a 500 kN load cell, displacement speed of 120 mm/min, and 100 mm claw distance. While testing with dry and hydrated CS woven mesh a 500 kN load cell, a speed of 100 mm/min, and a distance between claws of 40 mm were used. CS woven meshes samples were immersed in PBS solution at 37 ± 0.5 °C for 24 h. The analyzes were conducted at 25 ± 1 °C and relative humidity of 60 ± 2%. A universal testing machine was used (Instron Model 6633—Norwood, MA, USA).

### 2.7. Swelling Test

The swelling behavior of CS woven meshes (*n* = 3 to each time; 10 × 10 mm^2^) was evaluated in phosphate buffer saline (PBS, pH = 7.34) solution at 37 ± 0.5 °C. The samples were dried at 50 °C for 6 h, weighed (*W*_0_), and conditioned at 37 ± 0.5 °C in PBS solution. Samples were taken from the medium at predetermined periods (0.5, 1, 5, 10, 20, and 24 h), wiped with absorbent paper, and weighed (*W*_t_). The swelling behavior was evaluated by specific (measured in grams, mass variation, Δm) and relative mass variation (measured in percentage, Swelling Degree, SD_t_) determined according to the following equations:

(1) $$\Delta m\;\left(g\right)=W_{t}-W_{0},$$

(2) $${\mathrm{SD}}_{t}\left(\%\right)\;=\frac{\Delta m}{W_{0}}\times 100,$$

where *W_t_* and *W*_0_ represent the weights of swollen and dried state samples, respectively.

### 2.8. In Vitro Enzymatic Degradation

In vitro degradation of CS woven meshes (*n* = 3 for each time period; 10 × 10 mm^2^) was evaluated in 10 mL phosphate buffer solution (PBS, pH = 7.34) at 37 ± 0.5 °C containing 1.5 µg/mL lysozyme [24]. The samples were dried at 50 °C for 6 h, weighed (*W*_0_), and conditioned at 37 ± 0.5 °C in PBS/Lysozyme solution. PBS/Lysozyme solution was changed weekly to maintain enzyme activity. At each biodegradation period (7, 14, 21, 28 and 35 days), the samples were taken from the solution, washed in distilled water, pre-dried with absorbent paper, dried at 50 °C for 6 h, and then weighed (*W*_t_).

(3) $$Mass\;Loss\;\left(\%\right)=\frac{W_{0}-W_{t}}{W_{0}}\times 100,$$

where *W*_0_ is the initial mass of the sample, and *W_t_* is the mass of samples degraded at time *t*.

### 2.9. Statistical Evaluation

T-test and One-Way Analysis of Variance (ANOVA) analysis were performed using Minitab v19.1. The significant differences between conditions were determined by Fisher′s tests. The confidence level adopted was 95%, and the significance level (α) was 0.05. For *p*-values less than or equal to α, the difference between means was considered statically significant. For *p*-values greater than α, the difference between means was considered statically non-significant.

## 3. Results and Discussion

### 3.1. Threads Characterization

#### 3.1.1. Morphological Analysis

**Figure 3:**
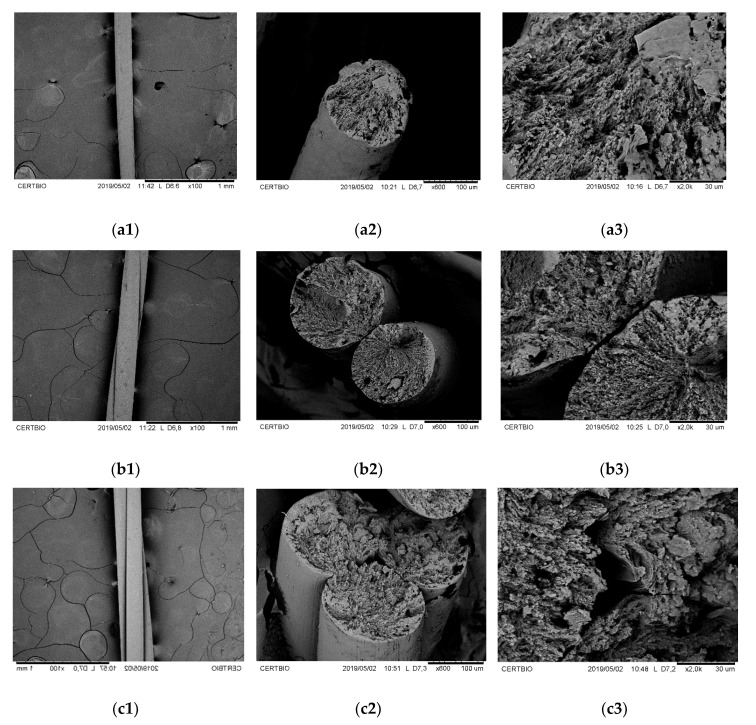
SEM micrographs of the chitosan threads longitudinal and cross-section: (**a1**–**a3**) Monofilament configuration; (**b1**–**b3**) two-filament configuration; and (**c1**–**c3**) three-filament configuration.

Scanning electron microscopy (SEM) analysis was used to evaluate the surface and cross-section of CS threads after cryogenic fracture and gold coating. SEM micrographs are presented in Figure 3.

In longitudinal surface micrographs (Figure 3a1, Figure 3b1 and Figure 3c1), was observed a smooth and compact surface, with well-defined cylindrical filamentary structures. In the smallest magnification micrographs of the cross-section (Figure 3a2, Figure 3b2 and Figure 3c2), it is possible to confirm that individual filaments showed a “skin-core” structure typical of filaments obtained by wet spinning [25]. During the coagulation step of the wet spinning process, the filament skin has formed the instant they come into contact with the coagulation bath, while the nucleus has time to relax. This phenomenon results in a microstructure where the surface polymeric chains are oriented axially and in a compacted way, while the nucleus chains are packaged inefficiently, resulting in internal pores observed in the SEM micrographs of the CS threads [26,27]. CS multifilament threads preserved the structure of single filament thread, compacted external surface, and porous nucleus. Table 1 presents the average values of diameter, twist angle, porosity, and average pore size for different configurations of CS threads. Where letters “A”, “B”, and “C” represent Fisher’s groupings for 95% confidence (means that do not share a letter are significantly different).

Chitosan (CS) threads diameters increasing with the number of filaments present. CS thread composed of multiple filaments presents a twist angle between 3.7 and 5.3°, a reduction of 1.6° occurs when the filament number decreases from 3 to 2. Compared to the monofilament CS thread, the porosity increases with the twist of two filaments (from 1.1 to 2.7%). However, there was a decrease in porosity when comparing multifilament CS threads, the porosity varies from 2.7 (two filaments) to 1.8% (three filaments), which may be related to a higher degree of torsion for than CS thread bi-filament. The average pore size did not vary significantly (by Fisher’s test) with CS thread configuration.

#### 3.1.2. Mechanical Properties

**Figure 4:**
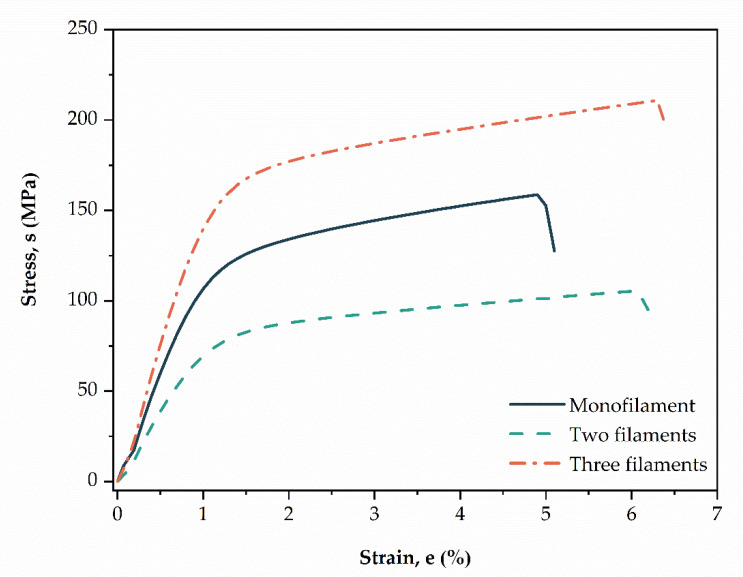
Representative strain-stress curves (*n* = 10) of the mono- and multifilament chitosan threads.

Figure 4 shows a representative stress vs strain graphic for CS threads obtained by uniaxial tensile test, and Table 2 summarizes the mean mechanical properties of the samples. Observed that monofilament CS thread showed intermediate mechanical properties between multifilament threads. This is confirmed in Table 2 with average values, where thread composed of two filaments showed the smallest values of tensile strength and elastic module. On other hand, the configuration with three filaments showed the highest values for the same properties. This may be due to the fact of thread with two filaments presenting greater porosity and pore size, due to the greater torsion angle (see Table 2), which caused the thread to become weak. Torsion of the CS filaments contributes to formed pores in the structure of threads, leading to uneven load distribution through the thread cross-section, causing a reduction in tensile strength [28,29]. This effect was also observed in our study previously reported [23].

Furthermore, one-way ANOVA and Fisher’s tests showed that none of the CS threads differ statistically in mean values of strain (*p*-value = 0.140). The mean values of tensile strength and the elastic module were statistically significant for different CS thread configurations (*p*-value < 0.000). In Table 2 the letters “A”, “B”, and “C” represent groupings according to the Fisher LSD test for 95% confidence, which means that do not share a letter are significantly different.

### 3.2. Woven Meshes Characterization

#### 3.2.1. Morphological Analysis

CS woven meshes were coded according to the thread configuration used in weaving as showed in Table 3.

**Figure 5:**
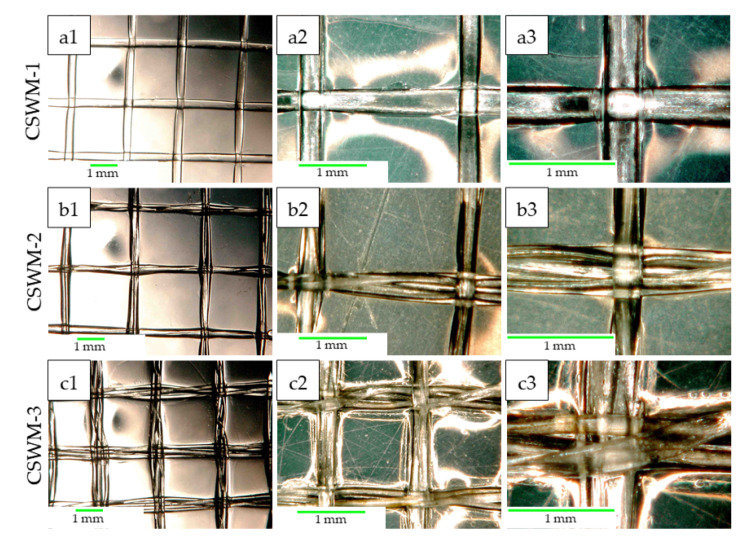
Optical microscopy (OM) micrographs of the chitosan woven meshes obtained by weaving: (**a1–a3**) Monofilament composed; (**b1–b3**) two-filament composed; and (**c1–c3**) three-filament composed.

Figure 5, shows the optical micrographs of CS woven meshes obtained by weaving of mono- and multifilament CS threads. It’s observed in the micrographs obtained in transmission mode (Figure 5a1, Figure 5b1 and Figure 5c1) that coating with 1.5% CS solution was homogeneous throughout the samples, and orthogonal weave between the CS was maintained for all configurations. Greater magnifications Figure 5(a2,a3,b2,b3,c2,c3) showed the configurations of the woven CS threads, where it is observed that the multifilament threads preserve the twisted structure. ImageJ software was used to measure the spaces in CS threads weaves (#). Monofilament CS woven meshes presented a # = 4.87 ± 0.30 mm^2^, to bi-filament, and tri-filament CS woven meshes the value of “#” was 4.18 ± 0.39 and 3.86 ± 0.37 mm^2^ respectively. As noted by Aghaei-Ghareh-Bolagh et al. [14], the # value of woven meshes is determined by the diameter of the threads, diameter, and distance between the loom pins. In this study, since the diameter and distance between the pins were not varied, the # value could be controlled from the diameter of the CS thread used in weaving, as shown in Table 1. The value of “#” does not differ statistically for samples CSWM-2 and CSWM-3 (*p*-value = 0.179), according to Fisher’s test.

#### 3.2.2. Mechanical Properties

Mechanical behavior in a hydrated state is more important than a dry state to biomaterials applications, due to exposure to physiological fluids [30]. Thus, the mechanical properties of the CS woven meshes were evaluated by uniaxial tests in dry and wet states.

**Figure 6:**
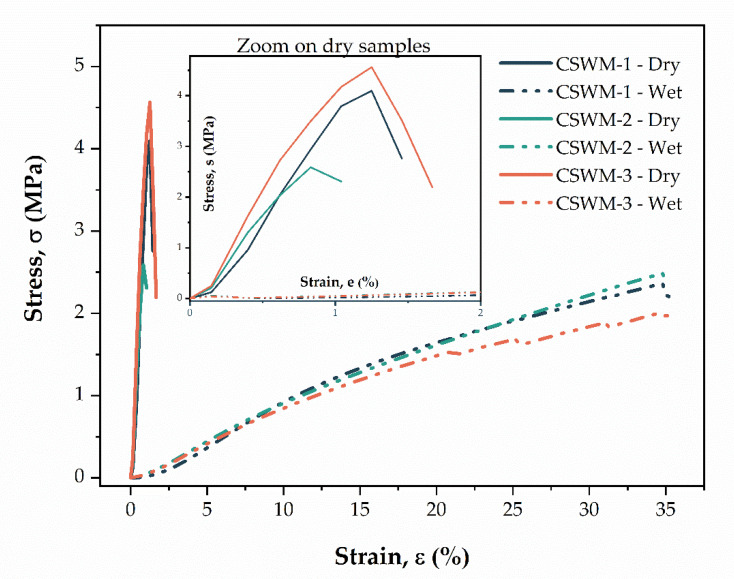
Representative stress–strain curves (*n* = 10) of the dry and hydrated of the chitosan woven meshes.

Figure 6 shows representative (*n* = 10) stress–strain curves for dry and hydrated CS woven meshes. Under elongation, the dry CS woven meshes exhibited a narrow elastic region with a high slope of the σ-ε curve, followed by failure at approximately 1% strain. On the other hand, hydrated samples showed a larger elastic zone of strain and lower slope of the σ-ε curve, with failure at approximately 35% strain.

**Figure 7:**
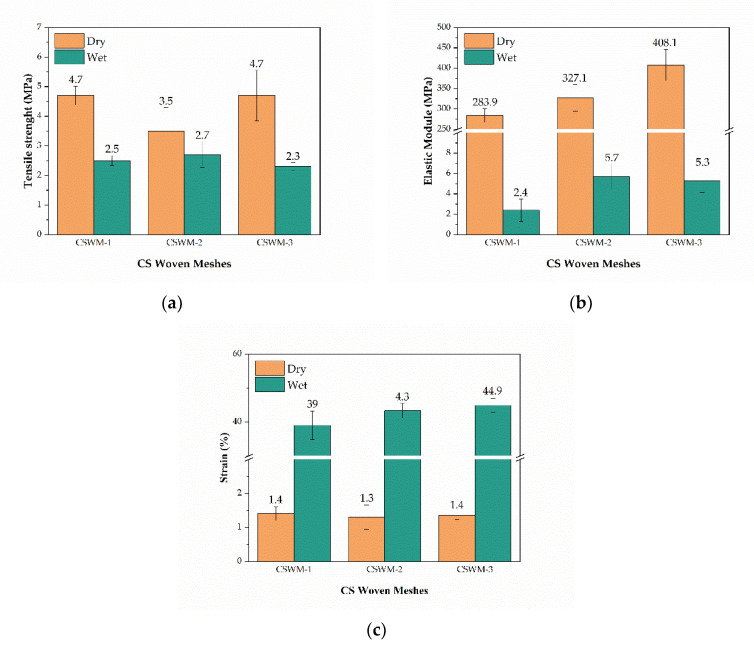
Comparison of (**a**) tensile strength, (**b**) elastic module, and (**c**) strain at break of dry and hydrated chitosan woven meshes (mean ± standard deviation).

Figure 7 shows the average mechanical properties for the CS woven meshes with different thread configurations. In dry conditions, the tensile strength of CS woven meshes was affected by thread configurations, with the CSWM-2 sample presenting a statistically significant reduction (*p*-value = 0.001). To elastic module was seen an increase with the number of filaments in CS threads weaving, however, only the CSWM-3 sample shows the statistical difference (*p*-value = 0.001). Furthermore, was not observed influence (*p*-value = 0.837) of thread configuration on the strain of the CS woven meshes.

In a wet state, CS woven meshes mechanical properties were not affected by the configuration of threads used in weaving, since mean values did not present statistical significance according to Fisher′s test (*p*-value > 0.05).

The tensile strength and elastic module of the CS woven meshes decreased from the dry to the hydrated state for all samples. The reduction of tensile strength was 46.8%, 22.8%, and 51.1% for CSWM-1, CSWM-2, and CSWM-3 samples, respectively. Elastic modulus showed a greater reduction than tensile strength in the order of 98 to 99% for all samples. However, deformation capacity and elasticity of the CS woven meshes were increases on average 30 times for all samples, as could be seen in strain values and the σ-ε curve. All of the mechanical properties variations to dry and wet states were statistically significant (*p*-value < 0.000).

Increases in the strain of the CS woven meshes in a hydrated state probably due to water molecules absorbed by samples. This interferes with intermolecular hydrogen bonds of the CS chains, causing an effect similar to that of a plasticizer additive. The interactions between polymer chains are reduced, facilitating the sliding of the chain about the other [31,32,33]. The reduction in tensile strength and elastic modules under wet conditions is in line with results reported in the literature for CS-based materials [14,34,35,36]. Furthermore, all CS woven meshes tensile strength similar to that of the native human dermis [37].

#### 3.2.3. Swelling Tests

**Figure 8:**
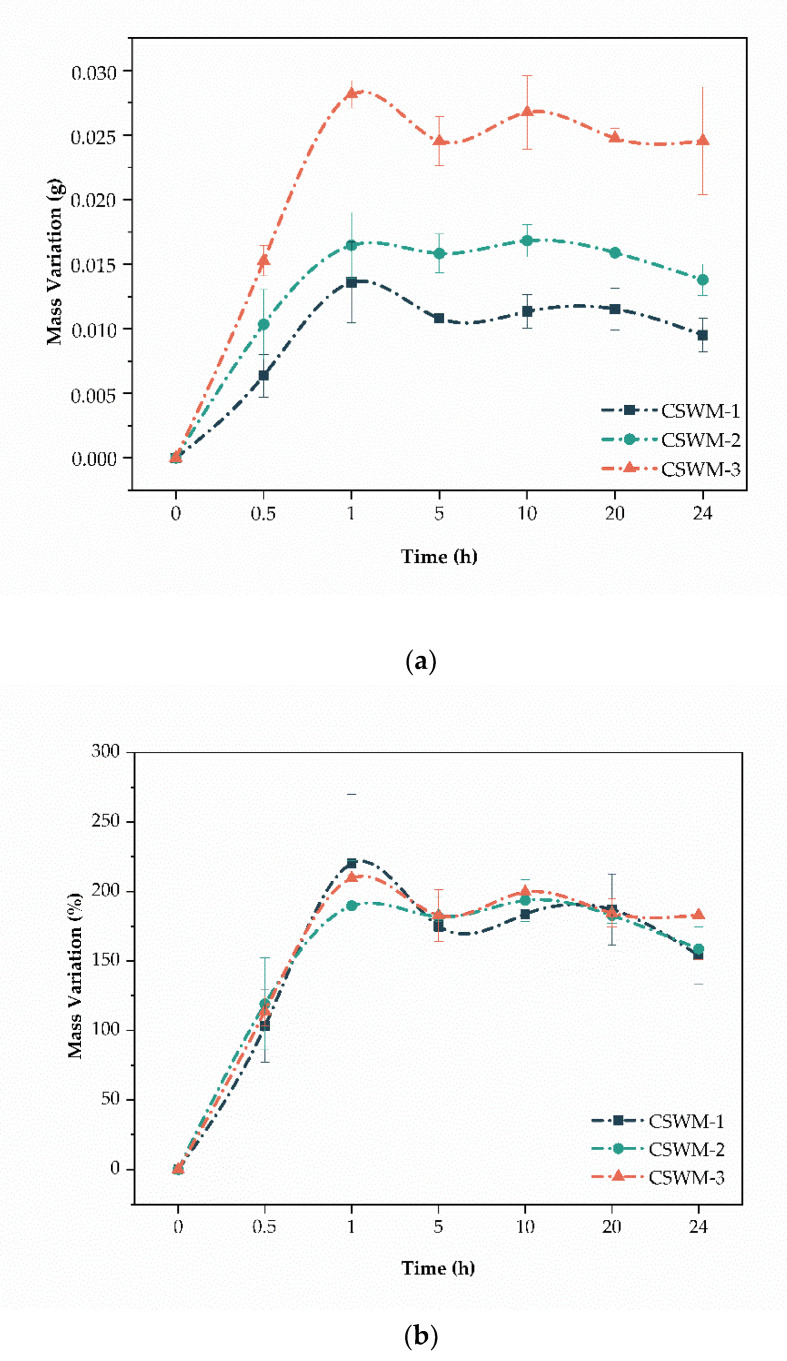
(**a**) Swelling behavior (absolute mass variation, in grams) of the chitosan woven meshes (mean ± standard deviation) and (**b**) swelling behavior (relative mass variation, in the percentage of the initial mass) of the chitosan woven meshes (mean ± standard deviation).

Swelling of the CS woven meshes was evaluated in PBS solution (pH = 7.4) at 37 ± 1 °C based on variation in absolute mass (g) and percentage (g) of the samples in periods of 0, 0.5, 1, 5, 10, 20, and 24 h. Figure 8a shows absolute mass variation and Figure 8b shows percentage mass variation. In absolute variations, samples composed of multifilament threads showed greater variation in mass, with the CSWM-3 sample being one with the greatest swelling. This is due to the greater number of filaments per unit area in the multifilament CS woven meshes. The swelling of CS-based materials in aqueous solutions is due to the formation of hydrogen bonds between absorbed water molecules and hydroxyl (OH) and free amino (–NH_2_) groups of CS [38,39]. Thus, the amount of fluid absorbed is proportional to the availability of these groups, that is, the amount (mass) of chitosan in the sample. Which justifies the biggest swelling of samples composed of multifilament threads.

In percentage variation, the samples showed similar swelling profiles, not statistically differing (*p*-value > 0.05) in all studied points. The maximum degree of swelling was reached in 1 h, and after that, it oscillated around the balance swelling (180.6 ± 12.9%). This is the result of the balance of osmotic forces determined by hydrophilicity and elasticity of polymeric network [7]. These results are in agreement with results found in the literature for chitosan-based materials [7,39,40].

#### 3.2.4. In Vitro Enzymatic Degradation

**Figure 9:**
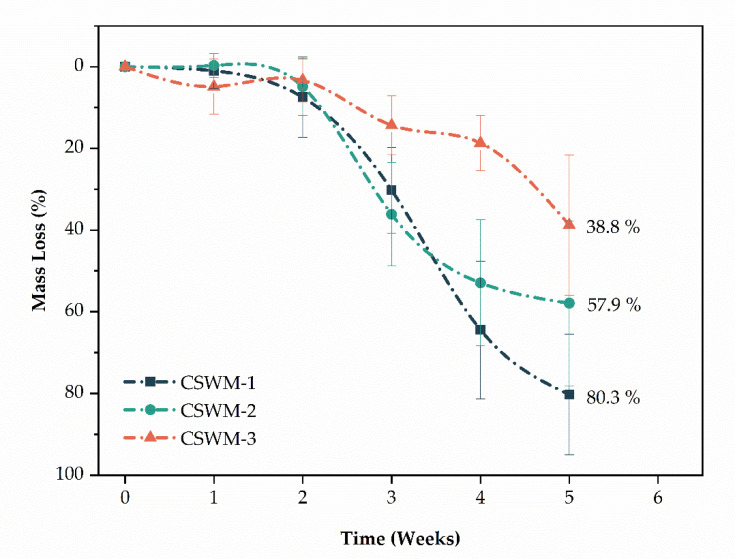
Mass loss of the chitosan woven meshes as a function of the degradation time (mean ± standard deviation).

In vitro enzymatic degradation of the CS woven meshes was evaluated by mass loss for a 5 weeks (35 days) incubation period in PBS solution containing lysozyme (pH = 7.34), in a concentration of 1.5 µg/mL, at 37 ± 0.5 °C [24]. Lysozyme is primarily responsible for the breakdown of CS in the human body. The in vitro tests is generally assessed using hen egg-white lysozyme [41,42,43], since it cleaves chitosan in its subunits, *N*-acetyl-glucosamine and glucosamine [44], facilitating its absorption and/or elimination through normal metabolic pathways [2]. Figure 9 shows the values of mass loss for CS woven meshes samples.

In up to two weeks, only mass fluctuations occur, caused by the swelling process of the samples, and interactions between degradation fluid, and amorphous and crystalline regions of the samples. Mass loss begins after two weeks for all of the CS woven meshes samples and proceeds in an accelerated way for CSWM-1 and CSWM-2 samples. After 4 weeks it is possible to observe a clear influence of thread configuration on degradation rates of the CS woven meshes. In mass losses were 80.3 ± 17.8% to CSWM-1, 57.9 ± 20.2% to CSWM-2, and 38.8 ± 17.2% to CSWM-3 samples. CSWM-2 sample showed no significant difference in relation to other samples (*p*-value > 0.05). However, difference between CSWM-1 and CSWM-3 samples was significant (*p*-value = 0.005). In this way, the enzymatic biodegradation of CS woven fabrics was modulated based on the configuration of the CS thread used in the weaving process.

The rate of biodegradation is a determining feature in the correct application of biomaterials; thus, the observed results may suggest different applications for CS woven meshes. For example, it can be said about enzymatic degradation profiles that the woven fabrics produced with different thread configurations may present different drug release profiles when applied as controlled drug delivery systems, adapting themselves to different treatment needs [2,45]. Furthermore, in wound healing the breaking of chitosan structure in its subunits, *N*-acetyl-glucosamine and glucosamine facilitate its absorption [44].

Glucosamine and *N*-acetyl glucosamine, are naturally occurring amino-monosaccharides that make up glycoproteins, proteoglycans, glycosaminoglycans and other connective tissue building blocks, in addition to acting as biochemical precursors for all amino-sugars of the human body [46,47,48]. In addition to therapeutic effects in reducing inflammation and accelerating the wound healing process [24,49,50]. This effect suggests that meshes woven from chitosan may act as bioactive dressings.

## 4. Conclusions

CS woven meshes have successfully obtained from weaving mono- and multifilament CS threads. Showing that the CS yarns obtained by wet spinning followed by twisting were suitable for the weaving process. The configuration of threads determined its morphological properties and consequently mechanical behavior, and the twisting of filaments was decisive for the internal porosity of threads.

Thread configuration was also significant for the morphology and dry mechanical properties of CS woven meshes; however, wet properties have not been affected drastically. Physiological properties, rate of biodegradations, and swelling behavior were also directly affected by the thread configuration used in weaving. Thus, the CS woven meshes obtained showed modulable properties based on the configuration of yarn used in weaving, suggesting potential applications in the biomedical field, as dressings, or as a controlled drug delivery system.

## Figures and Tables

**Table 1:** Parameters of the mono- and multifilament chitosan threads obtained from SEM analysis (mean ± standard deviation).

Thread Configuration	Diameter (µm)	Twist Angle (°)	Porosity (%)	Average Ore Size (µm)
Monofilament	171 ± 2 (A)	–	1.1 ± 0.89 (A)	0.66 ± 0.257 (A)
Two-filaments	260 ± 13 (B)	5.3 ± 0.30 (A)	2.7 ± 1.75 (B)	0.82 ± 0.230 (A)
Three-filament	312 ± 22 (C)	3.7 ± 0.92 (B)	1.8 ± 0.57 (A, B)	0.72 ± 0.274 (A)

**Table 2:** Mechanical properties of the mono- and multifilament chitosan threads obtained by uniaxial tensile testes (mean ± standard deviation).

Thread Configuration	Tensile Strength (MPa)	Elastic Module (Gpa)	Strain (%)
Monofilament	171 ± 22.26 (A)	8 ± 0.93 (A)	7 ± 2.23 (A)
Two-filament	127 ± 7.82 (B)	6 ± 0.26 (B)	8 ± 2.29 (A)
Three-filament	230 ± 24.38 (C)	11 ± 0.72 (C)	10 ± 2.3 (A)

**Table 3:** The nomenclature used for CS woven meshes.

Woven Meshes Code	Thread Configuration
CSWM-1	Monofilament
CSWM-2	Two-filament
CSWM-3	Three-filament
